# Comparative Analysis of Fluorescent Characteristics of Different Provisional Restorative Materials for Improved Dental Esthetics

**DOI:** 10.3390/polym16223184

**Published:** 2024-11-16

**Authors:** Pitchaya Aneksomboonpol, Awiruth Klaisiri, Awutsadaporn Katheng, Katanyoo Limchaikul, Papichaya Intajak, Nuttaphon Kittikundecha, Wisarut Prawatvatchara

**Affiliations:** 1 Department of Conservative Dentistry and Prosthodontics, Faculty of Dentistry, Srinakharinwirot University, Bangkok 10110, Thailand; pitchaya.pcy@g.swu.ac.th (P.A.); katanyoo@g.swu.ac.th (K.L.); papichaya@g.swu.ac.th (P.I.); 2 Division of Restorative Dentistry, Faculty of Dentistry, Thammasat University, Pathumthani 12120, Thailand; dentton@hotmail.com; 3 Department of Restorative Dentistry, Faculty of Dentistry, Naresuan University, Phitsanulok 65000, Thailand; awutsadaporn.k@gmail.com; 4 Department of Prosthodontics, Faculty of Dentistry, Chulalongkorn University, Bangkok 10330, Thailand

**Keywords:** provisional restorative materials, fluorescence, spectrophotometer, CIEDE2000

## Abstract

At present, provisional restorative materials are widely used for both short- and long-term treatment purposes. In esthetic dentistry, for the simulation of natural teeth, fluorescence plays a crucial role in enhancing the elegant, vibrant, and life-like appearance of restorative teeth, making them more closely resemble natural teeth. To achieve ideal esthetic outcomes, restorative materials must replicate the characteristics of natural teeth to provide the most realistic appearance possible. Thus, this study aims to compare the color difference in fluorescence (∆E_00_) between a normal bovine tooth and eight provisional restorative materials under ultraviolet radiation. Eight provisional restorative materials were fabricated, for a total of 80 specimens (size 13.0 mm × 8.0 mm × 1.0 mm). A sound bovine tooth incisor was collected and used as a control for both enamel and dentine conditions. The color difference in fluorescence (∆E_00_) was assessed using a spectrophotometer. A one-way ANOVA and Tukey’s test (*p* < 0.05) were used to analyze the resulting data. The results revealed that the color difference in fluorescence (∆E_00_) between the normal bovine tooth and eight provisional restorative materials used in this study was higher than the 50% acceptability threshold, indicating a clear mismatch between the fluorescence of the materials and that of the normal bovine tooth. Unifast Trad had the lowest ∆E_00_ among the provisional restorative materials, while Luxatemp Fluorescence had the highest color difference in fluorescence (∆E_00_). The color difference in fluorescence (∆E_00_) between Unifast Trad and Luxatemp Fluorescence is statistically significant. However, no significant difference was observed between Temporary CB, Vipi Block Trilux, and Protemp 4, or between Luxatemp Star and Luxatemp Fluorescence. It can thus be concluded that the ∆E_00_ mostly differs among the different materials. Luxatemp Star and Luxatemp Fluorescence exhibited the highest ∆E_00_ by a significant margin compared to the other groups.

## 1. Introduction

Provisional restorative materials are essential in fixed prosthodontic therapies. Interim restorations must adhere to patient health criteria throughout prolonged treatment periods due to unanticipated circumstances, such as laboratory delays, patient unavailability, and required gingival or temporomandibular joint therapies. The optimal interim fixed repair must safeguard the underlying preparation, pulp, and gingiva while facilitating the recovery of any affected soft tissues during the fabrication of the final restoration at the laboratory. It must fulfill the interconnected biological, mechanical, and esthetic criteria, including fracture resistance, marginal fit, color stability, wear resistance, tissue compatibility, simplicity of manipulation, and cost-effectiveness. [1,2]. For the goals of provisional treatment, it is necessary for the mechanical characteristics, such as occlusal mastication, as well as the physical attributes, such as shade and color, to remain consistent during the treatment period. The ease with which they can be adjusted and repaired throughout the course of the therapeutic process is another advantage.

The ability of a material to emit light after absorbing radiant energy is called photoluminescence. This occurs when an electron in a molecule or atom absorbs energy and moves to a higher energy state. When the electron returns to a lower energy state, it releases this energy as light, creating a glowing effect. When light with a specific wavelength interacts with a molecule, the molecule’s electrons absorb the energy. This causes the electrons to move from their normal, low-energy state (S0) to a high-energy, excited state (S1). This process is called excitation. Electrons only stay in the excited state for a short time and lose some energy during this period. When they return to their normal state (S0), they release the remaining energy as light. Photoluminescence can be divided into two types: phosphorescence and fluorescence [3]. The most crucial factor in stimulating photoluminescence phenomena is electromagnetic waves in the UV radiation range. In both phosphorescence and fluorescence, light is emitted, but the energy of the light is lower than the energy absorbed. Fluorescent materials glow immediately when exposed to ultraviolet (UV) radiation, but they stop glowing as soon as the light source is removed. Phosphorescent materials, however, release the absorbed energy more slowly, so they continue to glow for a while even after the removal of the radiation source.

Fluorescence was first described by the British scientist George Gabriel Stokes in 1852. He coined the term while studying the blue-white glow of the mineral fluorite (fluorspar) under ultraviolet (UV) radiation. Fluorescence is related to a key concept called the Stokes shift, which refers to the difference in wavelengths between the light that a fluorescent molecule absorbs and the light it emits. Fluorescence occurs when a material emits light with a longer wavelength than the light it absorbs. Electrons normally exist in a stable ground state, which is the lowest energy level. When they absorb energy, they move to a higher energy state, called the excited state, which has more energy than the ground state. To return to the ground state, electrons release this extra energy, often in the form of light (photons). Fluorescent materials only emit light when exposed to a specific wavelength of radiation, which most occurs at UV radiation, and excited electrons stay in the higher energy state for only a short time before returning to the ground state [4]. When electrons return to their ground state from a higher energy level, they release energy in the form of light. This light has a longer wavelength and less energy than the absorbed energy.

In the field of dentistry esthetics, achieving accurate hue and translucency matching is essential for replicating the appearance of realistic teeth under different lighting conditions. Natural teeth exhibit fluorescence due to the absorption of ultraviolet radiation (350–400 nm) by luminescent components in tooth tissues, which is then released in the visible spectrum at longer wavelengths (410–500 nm), making teeth appear bluish-white when exposed to ultraviolet radiation [5]. Enamel and dentine are both luminous tissues, although dentine’s fluorescence is higher than enamel because of its high organic material content [6]. It was believed that these tissues glow because of their organic content; that is, amino acids such as pyrimidine, pyridinoline, tryptophan, tyrosine [7,8,9], hydropyridines, dityrosine, and a complex of hydroxyapatite [10]. Factors that also affect the fluorescence of teeth are aging, bleaching, temperature, and defects, such as caries, stains, and discoloration. There is a consensus that dentine fluorescence can be caused by large organic compounds. Quantitative studies have been conducted to examine the sequential changes in dentine fluorescence. The nature of this autofluorescence remains a topic of controversy [8,10,11].

Restorative materials must precisely match the specified requirements for esthetic appearance to achieve the best esthetic outcomes, ensuring that the restoration blends seamlessly with the natural dentition and meets patient expectations for visual appeal [12]. However, under ultraviolet radiation conditions, it has been observed that many restoration materials are unable to replicate the fluorescence properties of natural teeth, which contributes to their overall appearance and vitality [13,14]. To address this challenge, several research studies have been conducted to explore and evaluate the impact of incorporating luminescent components into resin composite and ceramic materials. These studies aim to enhance the fluorescence characteristics of these restorative materials, potentially leading to improved esthetic results and a more natural appearance that more closely mimics the optical properties of natural teeth [15].

In recent times, resin composite and polymer materials have emerged as preferred materials for provisional purposes. This is not only because they can fulfill requirements but also because of the simplicity of their modification and repair throughout the treatment phase [2]. Recent advancements have led to the creation of dental resin composites and polymer materials that can be customized for specific applications. Restorations, ranging from temporary to semi-permanent or long-term restorations, have been accomplished with the use of indirect resin composites. Based on their compositions, polymethylmethacrylate (PMMA) and bis-acryl resins are increasingly being used for provisional restoration materials. PMMA was originally a provisional restorative material. The clinical advantages of PMMA include its ease of use and relining, as well as its affordability; its disadvantages include a strong odor, polymerization shrinkage, and exothermic polymerization. Subsequently, newer materials such as bis-acryl composite resins have been added to achieve better therapeutic outcomes. Methods that are commonly employed to create temporary dental restorations often involve several methods such as direct fabrication (chairside method), indirect fabrication (laboratory method), and pre-formed restorations. The clinical advantages of bis-acryl composite resins are ease of use, low polymerization shrinkage, and minimal pulpal irritation, while their disadvantages are higher costs and difficulties in performing alterations and repairs. Recently, the utilization of digital technology in dental care has grown in popularity. CAD/CAM (computer-aided design and computer-aided manufacturing) is one of the most transformative technologies in modern dentistry. Digital CAD/CAM manufacturing can be categorized into two main techniques: subtractive manufacturing using milling and additive manufacturing using 3D printing. In the manufacture of provisional restorations, subtractive manufacturing involves the milling of pre-polymerized resin blocks, while additive manufacturing uses 3D printing to construct the prosthesis layer by layer to achieve the desired shape [3,12,16,17,18,19,20]. The clinical advantages of milling are homogeneity, good surface hardness, and the absence of pulpal irritation; the disadvantages are high costs, the wastage of raw material, and the laboratory skill and time required. For printing, the clinical advantages are higher surface hardness, homogeneity, no pulpal irritation, and less wastage of raw material; the disadvantages are high costs and the laboratory skill and time required. While provisional materials have been investigated for mechanical qualities [21,22,23], accuracy [21,23,24,25], and color stability [26,27,28], there is a lack of research on the fluorescence characteristics of provisional restorative materials.

This article outlines the commonly used materials and examines the effects of fluorescence characteristics on various provisional restorative materials. The aim of this study is to thoroughly investigate and analyze the color differences in fluorescence exhibited by provisional restorative materials in comparison to the fluorescence characteristics of bovine teeth. In this study, bovine teeth are used instead of human teeth for several reasons, such as size and shape limitations, as well as challenges in sourcing human teeth. Moreover, bovine teeth are considered a good substitute because their fluorescence properties are similar to those of human teeth due to comparable physicochemical and structural characteristics. By undertaking this analysis, we seek to provide valuable insights into how these materials interact with light and their potential esthetic implications in dental applications. The null hypothesis posited for this research asserts that there is no significant difference in the color difference in the fluorescence (∆E_00_) of the various types of provisional restorative materials when compared with that of bovine teeth. This hypothesis serves as a critical benchmark against which the outcomes of our investigation are measured, allowing us to determine whether the provisional materials demonstrate any noteworthy variations in fluorescence compared with the natural tooth structure.

## 2. Materials and Methods

The calculation of the sample size using statistical software (G*Power v. 3.1.5, Faul, Erdfelder, Buchner and Lang, Heinrich Heine University, Düsseldorf, Germany) was based on a 5% margin of error and a 95% confidence level (significance of 0.05). The calculated total sample size was 32 from 8 groups. However, this study utilized a sample size of 10 per group to enhance parameter estimates and conform with previous investigations [29].

This study evaluated eight groups of commercial provisional restorative materials, namely Unifast Trad, Luxatemp Star, Luxatemp Fluorescence, Protemp 4, Nextdent C&B MFH, Optiprint Lumina, Temporary CB, and Vipi Block Trilux. A summary of these materials is provided in Table 1.

### 2.1. Specimen Preparation

A healthy bovine incisor (no caries, no discoloration, and no structural defects) was used to fabricate dentine substrates in this study. The bovine tooth was stored in 0.1% thymol at room temperature until required. The tooth was cut with a low-speed cutting machine equipped with a diamond disk (IsoMet 1000 Precision Cutter, Buehler, IL, USA) parallel to the long axis of the tooth to form rectangular-shaped dentine substrate specimens (13.0 mm × 8.0 mm × 1.0 mm). The rectangular-shaped dentine substrate specimens were finished and polished with 600-, 800-, and 1000-grit waterproof paper (NANO-1000S Single Wheel, Bench Top Grinder/Polishers with Timer, Tucson, AZ, USA). The final dimensions of the specimen were measured using a digital vernier caliper. The specimens were kept in a container and submerged in distilled water at 37 °C and 100% humidity.

**Figure 1:**
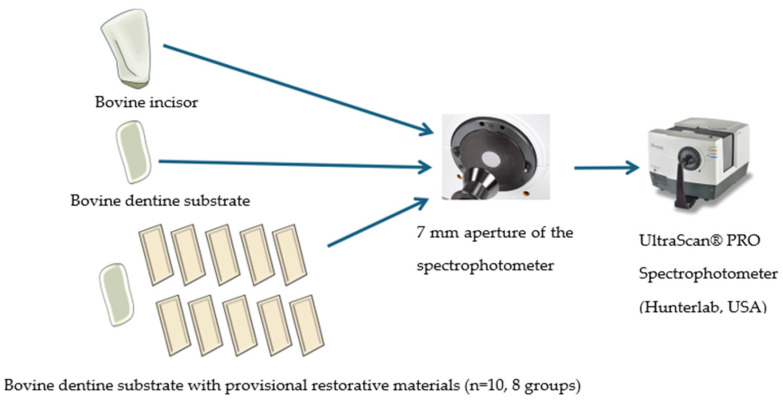
Study design and experimental method.

The materials used in this investigation included eight groups of commercial provisional restorative materials: Unifast Trad, Luxatemp Star, Luxatemp Fluorescence, Protemp 4, Nextdent C&B MFH, Optiprint lumina, Temporary CB, and Vipi Block Trilux. The experimental materials are summarized in Table 1. For the preparation of provisional restorative materials, two distinct methods were employed to ensure optimal results. The first method involved the traditional approach to creating conventional provisional restorative materials, which began with the use of a putty mold specifically designed to fit the contours of a bovine dentine substrate. After the initial setting of the material, the provisional restorations were then meticulously finished and polished using a series of abrasive disk papers, which included 600-, 8000-, and 1000-grit paper (NANO-1000S Single Wheel, Bench Top Grinder/Polishers with Timer, Tucson, AZ, USA), to achieve a smooth and refined surface finish for the provisional materials. The specimens were formed into rectangular shapes (13.0 mm × 8.0 mm × 1.0 mm). Second, for the preparation of digital provisional restorative materials, STL files (stereolithography, a format originally developed by 3D Systems for stereolithography from CAD software) were generated in rectangular shapes (13.0 mm × 8.0 mm × 1.0 mm) and forwarded to manufacturers for production. All specimens were kept in a container filled with distilled water at 37 °C and 100% humidity. The study design and the procedure of the experimental method are shown in Figure 1.

### 2.2. Fluorescence Measurement

Twenty-four hours prior to conducting the fluorescence measurement, a bovine tooth, along with all thirty-two specimens that had been categorized into eight distinct groups of provisional restorative materials, were carefully immersed in distilled water at room temperature. This step was essential to ensure that the specimens remained adequately hydrated and to prevent any potential dehydration.

For the fluorescence measurement, all specimens were tested with an UltraScan^®^ PRO Spectrophotometer (Reston, VA, USA) by a single operator to measure the fluorescence color under spectrophotometric analysis in accordance with CIEDE2000. The UltraScan^®^ PRO spectrophotometer has a spectral range of 350 nm to 1050 nm and offers three port diameters: a small area view (SAV) at 7 mm, a medium area view (MAV) at 13 mm, and a large area view (LAV) at 25 mm. For the fluorescence measurement in this study, the 7 mm SAV was used due to the size of the bovine tooth. Before the fluorescence measurements, the spectrophotometer was calibrated following the manufacturer’s guidelines for the 350–400 nm spectral range (ultraviolet-A). Spectrophotometric measurements were conducted with a white background. Each specimen was briefly removed from the distilled water and gently dried for no more than 2 min to prevent dehydration.

**Figure 2:**
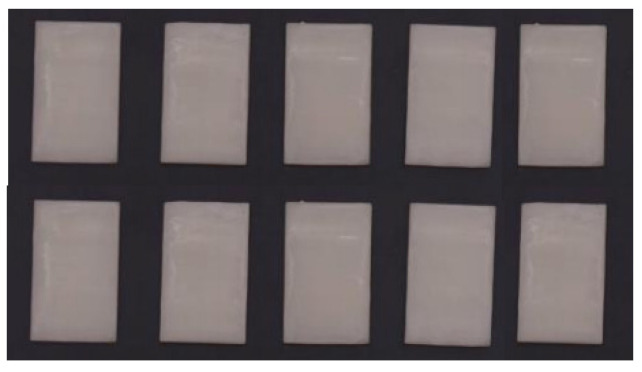
Samples of provisional restorative materials 10 pieces for one group.

**Figure 3:**
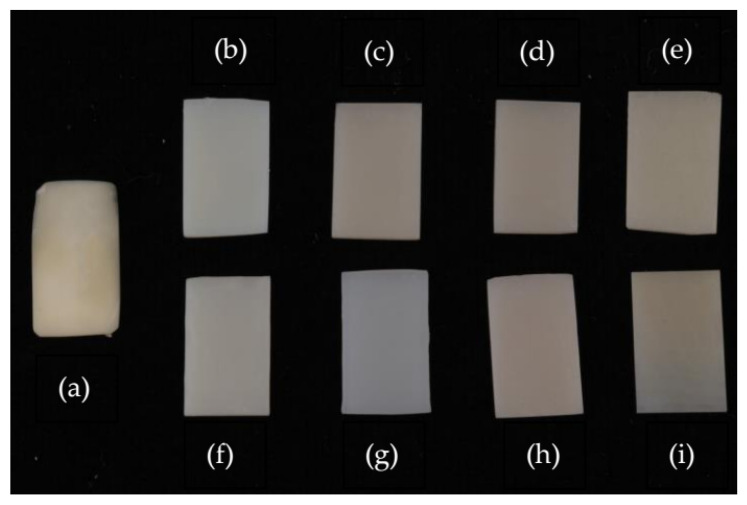
(**a**) Bovine dentine substrate, (**b**) Unifast Trad, (**c**) Luxatemp Star, (**d**) Luxatemp Fluorescence, (**e**) Protemp 4, (**f**) Nextdent C&B MFH (**g**) Optiprint lumina, (**h**) Temporary CB, (**i**) Vipi Block Trilux.

First, a healthy bovine incisor was measured with the spectrophotometer and then sectioned to create a dentine substrate for analysis. Each provisional restorative material (*n* = 10 per group) (Figure 2 and Figure 3) was then securely attached to the bovine tooth dentine substrate with glue pads on the sides and measured for fluorescence color against a white background.

All data were analyzed using HunterLab’s EasyMatch^®^ QC software version 4.98 to compare the differences in fluorescence color between the standard bovine incisor and dentine substrate with each group of provisional restorative materials. ∆E_00_ = {[∆L*/(k_L_S_L_)]^2^ + [∆C*/(k_C_S_C_)]^2^ + [∆H*/(k_H_S_H_)]^2^ + R_T_[∆C′/(k_C_S_C_)] × [∆H*/(k_C_S_C_)]}^1/2^, where ΔL*, ΔC*, and ΔH* represent the differences in lightness (ΔL*), chroma (ΔC*), and hue (ΔH*) between the two color specimens, respectively. S_L_, S_C_, S_H_: These are the weighing functions applied to the lightness (L*), chroma (C*), and hue (H*) components. They adjust the contribution of each component to the overall color difference based on perceptual factors. These functions help to account for the non-uniformity of the human visual system’s sensitivity to lightness, chroma, and hue. K_L_, K_C_, K_H_: These are parametric factors that are used to adjust the weighting functions (S_L_, S_C_, S_H_) according to specific viewing conditions or observer parameters. These factors allow for more accurate color difference calculation under different contexts, such as varying lighting conditions, display types, or other viewing environments. R_T_ is a correction term that accounts for interactions between chroma and hue differences, especially when the colors are highly saturated. It adjusts for situations where changes in chroma and hue together might cause a larger perceptual difference than would be expected from their individual components.

### 2.3. Statistical Analysis

A software program (SPSS 22.0 Windows, SPSS Inc., Chicago, IL, USA) was used for statistical analyses. For the mean data, the continuous outcome and normality of the distribution of the analyzed data were determined using the Shapiro–Wilk test at a significance level of 0.05. Levene’s test was then used to analyze the equality of variation. Then, a one-way analysis of variance (ANOVA) was used to analyze the data, followed by post hoc multiple comparisons with Tukey’s Honestly Significant Difference test (*p* < 0.05).

## 3. Results

**Figure 4:**
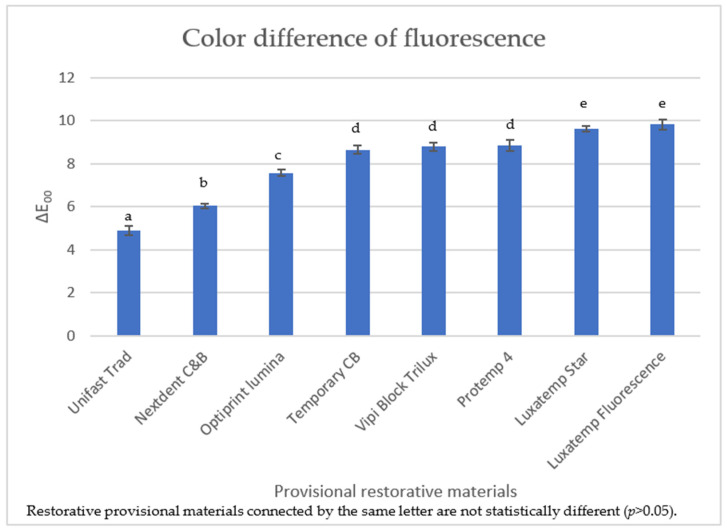
Bar chart showing the mean and standard deviation of the color difference in fluorescence (∆E_00_) of eight groups of provisional restorative materials. Restorative provisional materials connected by the same letter are not statistically different (*p* > 0.05).

Table 2 summarizes the average number and standard deviation of the color difference in the fluorescence (∆E_00_) of the provisional restorative materials. The ∆E_00_ results ranged from 4.5985 to 9.8292. Unifast Trad exhibited the lowest ∆E_00_, and Luxatemp fluorescence presented the highest ∆E_00_ of the provisional restorative materials under study. The provisional restorative materials were ranked by the lowest to highest ∆E_00_ values as follows: Unifast Trad, Nextdent C&B MFH, Optiprint lumina, Temporary CB, Vipi Block Trilux, Protemp 4, Luxatemp Star, and Luxatemp Fluorescence. No significant differences were observed in the color difference in fluorescence (∆E_00_) between Temporary CB, Vipi Block Trilux, and Protemp 4 or between Luxatemp Star and Luxatemp Fluorescence (*p* < 0.05). Bar chart of the color difference in fluorescence (∆E_00_) of eight groups of provisional restorative materials are illustrated in Figure 4. This indicates that, in terms of fluorescence color differences, the performances of these materials are quite similar. 

## 4. Discussion

In this study, the color difference in fluorescence (∆E_00_) of eight provisional restorative materials was examined using extracted bovine teeth and a bovine dentine substrate. The investigation revealed color differences in fluorescence between the provisional restorative materials and bovine dentine. Consequently, the null hypothesis was rejected, indicating that not only did the fluorescence intensities of the provisional restorative materials differ among groups, but they also differed from those of the extracted bovine teeth.

Bovine teeth were used as substitutes for human teeth in this study because it can be challenging to control the source and age of the collected human teeth, which may lead to larger variations in the outcome measures of the study [30]. Furthermore, the relatively small and curved surface area of human teeth may also be a limitation for specific tests requiring flat surfaces of a uniform thickness [30]. In this study, the 7 mm aperture size of the UltraScan^®^ PRO Spectrophotometer (Reston, VA, USA) was too large to accommodate human tooth specimens. However, several studies have reported that the fluorescence characteristics of bovine teeth closely resemble those of human teeth due to their similar physicochemical, structural, and biological features [31]. Therefore, bovine teeth were utilized in this study.

For esthetic appearance in dentistry, an understanding of the form, function, and appearance of natural materials is essential. Fluorescence is essential to esthetics and has a crucial function in the field of dentistry. Teeth can seem more authentic, vibrant, luminous, and genuine due to this optical characteristic. When exposed to ultraviolet radiation, natural teeth exhibit a bluish-white glow, which is referred to as the fluorescence effect [5]. Fluorescence is the ability of a substance to absorb electromagnetic waves, specifically in the ultraviolet radiation spectrum, and then emit them at longer wavelengths. Accurately imitating fluorescence is difficult when trying to achieve a precise tooth-like appearance owing to the many factors that affect esthetic characteristics. Provisional restorative materials must also enhance the esthetic look of replicated teeth, both in natural light and in ultraviolet light at night. Thus, manufacturers improve their provisional restorative materials to achieve fluorescence color properties by incorporating an accurate mixture of different components in particular proportions. These components include rare earth oxides such as samarium, dysprosium, cerium, terbium, ytterbium, and/or europium oxide [32,33].

In evaluations of fluorescence characteristics, a variety of methods and instruments have been employed to ensure accurate and comprehensive assessments. Among these methods, one can find the use of advanced fluorescence photography cameras, sophisticated fluoroscopic microscopes, versatile monochromator-based multimode microplate readers, precise spectrophotometers, and specialized ultraviolet illumination systems. Additionally, the fluorescence color can be assessed using several instruments, including fluorescence microscopes, fluorometers, photometers, fluorescence plate readers, and spectrophotometers.

Among these tools, fluorometers and spectrophotometers stand out as the most commonly utilized instruments in fluorescence studies, primarily due to their remarkable capability to measure fluorescence responses to ultraviolet radiation, specifically within the 350–400 nm wavelength range. This preference arises from their design, which is meticulously tailored to evaluate crucial parameters such as fluorescence intensity and color, thereby making them versatile and effective tools for a wide array of applications in fluorescence research and analysis [34]. Thus, the fluorescence of provisional restorative materials was determined with a color-measuring spectrophotometer according to the Commission Internationale de L’Eclairage (CIE) system.

This study revealed significant variance in the color difference in fluorescence based on the type of provisional restorative material used. Luxatemp fluorescence presented the highest color difference in fluorescence (∆E_00_) among the provisional restorative materials. In contrast, Unifast Trad presented the lowest color difference in fluorescence (∆E_00_) among the provisional restorative materials. However, upon conducting our analysis, we found no significant difference among the materials Temporary CB, Vipi Block Trilux, and Protemp 4. Similarly, there was also no significant difference observed between Luxatemp Star and Luxatemp Fluorescence in our study. The various fluorescence color results can be related to variations in the type and quantity of rare earth oxides [15]. However, the manufacturer does not provide any information regarding the specific type and quantity of rare earth oxides.

The color differences in fluorescence (∆E_00_) ranged from 4.5985 to 9.8292 for all provisional restorative materials evaluated in this study. Currently, the known clinical limitations of fluorescence emission are not under study. [35]. ∆E_00_ values of 1.8 were unacceptable in 50% of observers [36]. Therefore, the color difference in the provisional restorative materials used in this study was more than acceptable in 50% of observers, indicating a noticeable mismatch between the fluorescence of the materials and that of the natural tooth. This could result in the formation of various types and quantities of inorganic fillers and rare earth oxide loads, which are included in provisional restorative materials that differ from restorative resin composites. Additionally, several other critical factors can significantly impact the fluorescence effect of these materials. These include the thickness of the restorative material, the nature of the underlying substrate, the effects of aging on the material, variations in temperature during application or use, and the type and shade of the dental restorative material. Each of these elements can profoundly influence not only the esthetic outcomes but also the functional performance of the materials in a clinical setting, highlighting the complexity of achieving optimal results in dental restoration procedures [29,36,37]. Therefore, it would be highly advantageous to conduct a comprehensive investigation into the development of advanced provisional restorative materials that would more accurately replicate the fluorescence properties observed in natural teeth. Such an exploration would not only enhance the esthetic integration of these materials but could also improve overall patient satisfaction and clinical outcomes by ensuring a more seamless and natural-looking restoration.

One notable limitation of this study is that our examination of the fluorescence properties was exclusively confined to provisional restorative materials in shade A2 with a 1 mm thickness, without considering a broader spectrum of other shades and various thicknesses that could potentially yield different fluorescence characteristics. This narrow focus restricts the generalizability of our findings to other shades that might be used in clinical practice. Moreover, this study did not delve into the influence of temporary luting cements on the variations in fluorescence intensity observed between provisional restorative materials and bovine teeth. This aspect is particularly important, as the choice of luting cement could significantly affect the overall fluorescence response. Therefore, it is evident that further investigation is required to comprehensively understand these factors and their implications in dental restorative practices.

## 5. Conclusions

The color differences in fluorescence among the various provisional restorative materials that were examined consistently exceeded the generally accepted clinical threshold for color acceptability. As a result, the color differences in the fluorescence of the provisional materials differed significantly across the designated groups. Furthermore, when compared across all groups, Luxatemp Star and Luxatemp Fluorescence exhibited the highest color differences in fluorescence compared with the other provisional restorative materials. Therefore, the properties and characteristics evaluated in this study were significantly influenced by the materials tested. Depending on specific provisional requirements, some materials may be more suitable than others. Hence, a case-by-case analysis is essential when assessing the clinical performance of provisional restorative materials.

## Figures and Tables

**Table 1:** Detailed information of provisional materials.

	Product Name	Color	Manufacturer	Composition
PMMA	Unifast Trad	Ivory	GC America, Alsip, IL, USA	Polymethyl methacrylate, pigments, polymerized ethyleneglycol dimethacrylate
Bis-acryl composite resins	Luxatemp Star	A2	DMG, Hamburg, Germany	Bis-acrylic composite resin
	Luxatemp Fluorescence	A2	DMG, Hamburg, Germany	Bis-acrylic composite resin
	Protemp 4	A2	3M ESPE, St. Paul, MN, USA	Bis-acrylic composite resin, and a second functionalized dimethacrylate resin
3D printing resin	Nextdent C&B MFH	A2	Nextdent, Soesterberg, The Netherlands	Methacrylic oligomer, glycol methacrylate, phosphine oxide, inorganic filler, pigment
	Optiprint lumina	A2	Dentona AG., Dortmund, Germany	Methacrylic oligomer, inorganic fillers
	Temporary CB	A2	Formlabs Inc., Somerville, MA, USA	Esterification products of 4,4-isopropylidenediphenol, ethoxylated and 2-methylprop-2-enoicacid, diphenyl (2,4,6-trimethylbenzoyl)
Milling	Vipi Block Trilux	A2	Dentsply Siirona, Charlotte, NC, USA	Polymethylmethacrylate, polymerized ethyleneglycol dimethacrylate, organically modified ceramics, fluorescence, double cross link

**Table 2:** Mean and standard deviation (SD) of the color difference in fluorescence (∆E_00_) of healthy bovine incisor and dentine substrate with eight groups of provisional restorative materials (*n* = 10).

Provisional Restorative Materials	∆E_00_ (SD)
Unifast Trad	4.5985 (0.21) ^a^
Nextdent C&B MFH	6.0552 (0.11) ^b^
Optiprint lumina	7.5684 (0.14) ^c^
Temporary CB	8.6467 (0.19) ^d^
Vipi Block Trilux	8.7970 (0.20) ^d^
Protemp 4	8.8431 (0.25) ^d^
Luxatemp Star	9.6341 (0.21) ^e^
Luxatemp Fluorescence	9.8292 (0.24) ^e^

Restorative provisional materials connected by the same letter are not statistically different (*p* > 0.05).

## Data Availability

The original contributions presented in the study are included in the article; further inquiries can be directed to the corresponding authors.
